# Enhancing glycaemic control and promoting cardiovascular health: the therapeutic potential of *Trigonella foenumgraecum* in diabetic patients – a systematic review and meta-analysis

**DOI:** 10.1097/MS9.0000000000001750

**Published:** 2024-01-25

**Authors:** Saad Shakil, Sareema E. Akhtar, Akhtar Ali, Meetty Antony, Ishan Antony, Eyad Mansour, Sabeeh Khawar Farooqui, Absam Akbar, Hosam Alazazzi, Reema Alsufyani, Majid Alsufyani, Retaj Alawadhi, Rahul K. Ramtohul, Sebastian Hadeed, Aysa Tabassi, Aylin Tabassi, Talal Almas

**Affiliations:** a Internal Medicine, Ziauddin Medical College; b Department of Pharmacology, Ziauddin Medical University; c Aga Khan University Hospital, Karachi, Pakistan; d New Cross Hospital, Wolverhampton; e Morriston Hospital, Wales, UK; f Jawaharlal Nehru Medical College, Belgaum, Karnataka, India; g Royal College of Surgeons in Ireland, Busaiteen, Bahrain; h Beaumont Hospital; i Royal College of Surgeons in Ireland, Dublin, Ireland; j Lahey Hospital and Medical Center, Burlington, Massachusetts; k University Hospitals Cleveland Medical Center, Ohio, USA

**Keywords:** fasting blood glucose, fenugreek (*Trigonella foenumgraecum*), HbA1c, randomized clinical trials, type 2 diabetes mellitus

## Abstract

**Background:**

The antidiabetic potential of fenugreek has been highlighted in past literature, and various in-vitro and in-vivo studies have validated its glucose-lowering effects; however, very limited data are available on its effects on diabetic patients.

**Objective:**

An updated systematic review and meta-analysis of randomized control trials that assessed patients who were administered fenugreek.

**Methods:**

The PRISMA guidelines (Supplemental Digital Content 1, http://links.lww.com/MS9/A361) were followed when conducting this meta-analysis. PubMed, Scopus, Google Scholar and MEDLINE were searched from inception until June 2023, for randomized control trials that compared fenugreek with control in patients with type 2 diabetes mellitus (DM) and reported the following outcomes of interest: fasting blood glucose, glycated haemoglobin A1c (HbA1c) and postprandial glucose levels. The findings were presented as mean difference (MD) with 95% confidence intervals (CIs) and were pooled using a random effects model.

**Results:**

Fenugreek significantly (*P*<0.001) reduced the fasting blood sugar (FBS), HbA1c levels and postprandial glucose levels in diabetic patients when compared to the control.

**Conclusion:**

Among patients with type 2 DM, our comparisons demonstrated a reduction in FBS, HbA1c levels and postprandial glucose levels with the administration of fenugreek seed at 2–5 mg dose in powder form.

## Introduction

Highlights

- A meta-analysis of five clinical trials with a total of 626 diabetic participants showed that fenugreek seeds have significant antidiabetic effects, resulting in a reduction in fasting blood sugar (FBS) levels and glycated haemoglobin A1c (HbA1c) levels.
- None of the trials reported any side effects after administering fenugreek seed at various doses, suggesting its safety profile.
- The strength of the current analysis included an extensive literature search and review of currently reported trials, a focused approach to identify the trials reporting FBS and HbA1c levels after administration of fenugreek in powder form only and not in combination with other herbs or standard oral hypoglycaemic drugs, and studies were selected with the presence of randomization and a controlled group.
- Future studies should focus on identifying the phytoconstituents present in fenugreek seeds to determine their specific mechanisms of action, standardize the content of fenugreek herbal products, and evaluate adverse effects, drug interactions, and potential benefits for diabetes and obesity treatment. Additionally, more randomized control trials with larger sample sizes and longer durations are needed to assess the effects of fenugreek on HbA1c levels.

Diabetes is characterized by high blood glucose levels in the blood. The reason may be either insulin resistance or inadequate synthesis of insulin by the pancreas. The most common type of diabetes is type 2 diabetes mellitus (T2DM), which is more common in adults. Approximately, 422 million people residing in low-income and middle-income countries suffer from diabetes globally. Every year, diabetes directly contributes to 1.5 million fatalities. Both the incidence and prevalence of diabetes have steadily increased over the last several decades. According to the projections, there would be 578 million diabetics worldwide by 2030 and 700 million diabetics by 2045 compared to 463 million in 2019^1,2^.

The main strategy for controlling diabetes combines pharmacological therapies with dietary and lifestyle modifications^3^. The need for low-cost, non-toxic medicines arises from the adverse effects that come with prolonged usage of these medicines^1^. A category of healthcare known as complementary and alternative medicine (CAM) is herbal medicine. CAM is defined by the US National Center for Complementary and Alternative Medicine (NCCAM) as a group of various medical and healthcare systems, medicines, supplements and practices that are not a part of traditional or mainstream medicine^4^. A major percentage of the medications that are now on the market have either been directly or indirectly obtained from plant sources, which are a good supply of pharmaceuticals. More than 800 plants may exist, according to recent literature, and they may have anti-hyperglycaemic properties. However, one of the most significant and traditional herbs used in terms of safety and efficacy is fenugreek seeds (*Trigonella foenumgraecum*), which are currently being advertised as a nutraceutical with the potential claim of reducing blood glucose levels^5^.

Various in-vitro and in-vivo trials have documented the glucose-lowering effects of fenugreek^6^–^8^; however, limited clinical trials have been conducted to document its glucose-lowering and insulin secretory effects. Thus, the primary objective of conducting this meta-analysis was to assess the glucose-lowering efficacy of fenugreek seeds used in powder form in diabetic patients. The parameters evaluated in the meta-analysis include fasting blood sugar (FBS) levels, glycated haemoglobin A1c (HbA1c) and postprandial glucose levels. While fenugreek seeds have been the subject of studies in the past, our systematic review stands out since it focused solely on clinical trials using fenugreek seed powder. This narrow emphasis allows for a precise assessment of fenugreek’s effectiveness in controlling glycaemic indicators such FBS, HbA1c and postprandial glucose levels. Additionally, to ensure relevance and timeliness, our analysis includes the most recent data accessible until June 2023. With the use of these distinctive characteristics, we want to address the demand for precise, recent research in this field and offer a thorough and modern knowledge of the function of fenugreek in enhancing glycaemic management in people with T2DM.

## Methodology

### Data sources and strategy

This meta-analysis was conducted in line with the Preferred Reporting Items for Systematic Reviews and Meta-Analyses (PRISMA, Supplemental Digital Content 1, http://links.lww.com/MS9/A361) standards^9^ and Assessing the Methodological Quality of Systematic Reviews (AMSTAR, Supplemental Digital Content 2, http://links.lww.com/MS9/A362) guidelines^10^.

### Study selection

An elaborate search approach was used by two independent reviewers (S.S. and S.E.A.) to conduct an electronic search of PubMed from June 2012 to June 2023 that included all potential terms of ‘Fenugreek, Trigonella Foenumgraecum, Glucose, Diabetes and RCTs’ coupled with MeSH words and the ‘AND’ and ‘OR’ Boolean operators. This meta-analysis is an update to a previously conducted meta-analysis that included at least a 12-month search overlap with the last meta-analysis^11^. Detailed search methodology is presented in Table S1, Supplemental Digital Content 3, http://links.lww.com/MS9/A363. The predetermined eligibility requirements for our meta-analysis were: (a) randomized controlled trials (RCTs) that have been published; (b) adult patients (≥18 years) and paediatric patients; and (c) a patient population comprising of healthy overweight, T2DM and pre-diabetic participants. This study was limited to articles available in English only; however, there were no geographical limitations. The studies included in the analysis varied in duration and encompassed patients who were both overweight and non-diabetic. This approach was driven by the constraints posed by the existing literature and the primary objective of our study, which was to delve into potential applications of fenugreek beyond the conventional scope of managing established diabetes.

Discussion and agreement with a third investigator (A.A.) helped to address any disagreements between the two independent reviewers (S.S. and S.E.A.) over the selection of studies. All the relevant articles were enroled first based on title and abstract, and later full-text reading was given to assess for relevance.

### Data extraction and quality assessment of studies

Two separate reviewers (S.S. and S.K.F.) cross-checked the studies that our search approach revealed and collated them in Mendeley Reference Manager 2.76.0. The primary outcome with regard to the study was the efficacy of fenugreek compared to control in T2DM. The efficacy outcomes related to fenugreek in comparison to placebo were noted and exported to Excel spreadsheets after being extracted from shortlisted studies. Two reviewers independently extracted data and evaluated the quality of the meta-analysis (S.S. and S.E.A.). For the primary outcomes of ‘Fasting Blood Glucose’, ‘Hb1Ac’ and ‘Post-prandial glucose levels’, a continuous set of data was extracted. Additionally, the text’s Table 1 reports the study characteristics that were also retrieved and reported. To assess the quality of studies, the ROBINS-II risk of bias assessment tool was utilized to evaluate the calibre of the included studies^23^ (Table S2, Supplemental Digital Content 3, http://links.lww.com/MS9/A363).

**Table 1:** Baseline study characteristics

Study name, year and country	Population, medication	Design	Age (mean)	Sample size	Duration (days)	Fenugreek preparation	Daily dose (g)	Control	Outcomes
Verma *et al*., 2016, India^12^	Type 2 DM, metformin ± sulphonylurea	RCT	NR	154	90	Fenugreek capsule	0.5	Placebo	FBS, postprandial plasma sugar, HbA1c, fasting and postprandial C-peptide level, body weight, BP, pulse rate
Ranade and Mudgalkar, 2017, India^13^	Type 2 DM, OHAs and insulin	RCT	48.0	60	168	Fenugreek seeds	10	No placebo	FBS, HbA1c, anthropometric measures
Hadi *et al*., 2020, Iran^14^	Type 2 DM	RCT	47.7	48	56	Powder	5	No placebo	FBS, ALT, ALP, AST, BUN, Cr, SBP, DSP, eGFR
Sundaram *et al*., 2018, India^15^	Type 2 DM, metformin	RCT	NR	80	56	Powder	25	No placebo	FBS, serum lipids, TC, LDL, HbA1c, triglycerides
Hassani *et al*., 2019, Iran^16^	Type 2 DM, glycaemic drugs or insulin	RCT	51.23	62	56	Powder	5	Placebo	FBS, HbA1c, BMI, waist circumference, BP, quality of life
Lu *et al*., 2008, China^17^	Type 2 DM, sulphonylurea	RCT	54.26	69	84	Capsule	6.3	Chinese yam as placebo	FBS, PPG, HbA1c
Rafraf *et al*., 2014, Iran^18^	Type 2 DM, metformin + glibemclamide	RCT	40.54	44	56	Powder	10	Placebo	FBS, HbA1c
Suchitra and Parthasarathy, 2015, India^19^	Type 2 DM, oral hypoglycaemic agents	RCT	50.2	60	56	Seeds	30	No placebo	HbA1c
Gaddam *et al*., 2015, India^5^	Pre-diabetic, none	RCT	–	79	1095	Powder	10	No placebo	FBS, PPG
Gupta *et al*., 2018, India^20^	Type 2 DM, antidiabetic drugs	RCT	–	100	84	FENFURO capsule	–	No placebo	FBS, HbA1c
Gupta *et al*., 2001, India^21^	Type 2 DM, sulphonylurea + biguanide	RCT	51.0	25	60	Capsule	–	Placebo	FBS, PPG, HbA1c
Chevasses *et al*., 2010, France^22^	Healthy overweight, NA	RCT	38	39	42	Coated tablet	1.176	Placebo	FBS

ALT, alanine aminotransferase; ALP, alkaline phosphatase; AST, aspartate aminotransferase; BP, blood pressure; BUN, blood urea nitrogen; BMI, body mass index; DM, diabetes mellitus; DSP, diastolic blood pressure; eGFR, estimated glomerular filtration rate; FBS, fasting blood sugar; HbA1c, glycosylated haemoglobin A1c; LDL, low-density lipoprotein; NR, not reported; OHAs, oral hypoglycaemic agents; PPG, postprandial glucose; RCT, randomized control trial; SBP, systolic blood pressure; TC, total cholesterol.

### Statistical analysis

Using Review Manager (RevMan) Version 5.4 (Nordic Cochrane Center, Copenhagen, Denmark, The Cochrane Collaboration), the retrieved data were evaluated. With 95% confidence intervals (CIs), all of the key continuous outcomes were expressed as weighted mean differences (WMDs). The model with random effects was chosen due to the observed differences between the study populations and study settings. A *P*-value of 0.05 or less was regarded as significant. The WMD and related 95% CIs were used to display the results.

Furthermore, the heterogeneity of effect sizes was evaluated using Higgin’s *I*^2^ statistics, where an *I*^2^ value of more than 50% was regarded as significant^24^. *P*-value <0.05 was considered significant for all the above analyses^25^. The manuscript was registered on PROSPERO (Prospero ID: CRD42023443601).

## Results

The current meta-analysis included 12 clinical trials reporting the antidiabetic effects of fenugreek seeds; the total number of participants (diabetic patients) included in the trials was *n*=1718. The PRISMA flow chart (Fig. S1, Supplemental Digital Content 3, http://links.lww.com/MS9/A363) encapsulates the selection and study process. Our initial search of PUBMED yielded 251 articles. After the implementation of inclusion and exclusion criteria, data from 12 studies were collected. Characteristics of the 12 included studies (*n*=1718) comparing fenugreek to a control/placebo are included in Table 1. All the studies reported efficacy outcomes.

### FBS in T2DM patients

**Figure 1:**
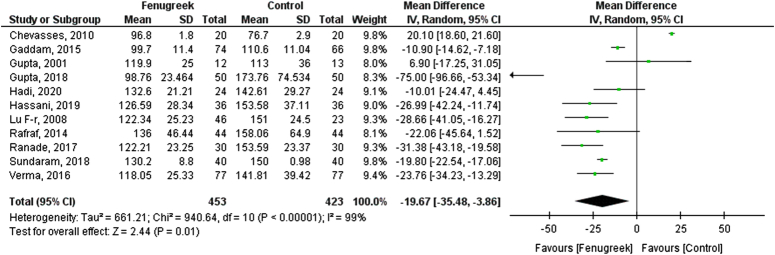
Fasting blood sugar (FBS) for fenugreek vs. control.

**Figure 2:**
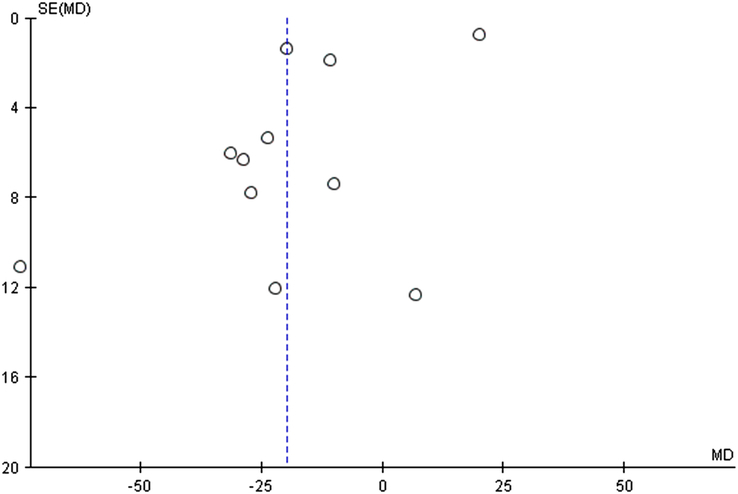
Funnel plot of fasting blood sugar (FBS) for fenugreek vs. control.

In our pooled analysis, 11 studies comprising 876 participants reported FBS, which was statistically significant. Fenugreek effectively reduced the levels of FBS [mean difference (MD) = −19.67 [−35.48, −3.86], *P*<0.00001, *I*^2^=99%; Figs 1, 2] when compared with vehicle in type 2 diabetic patients.

**Figure 3:**
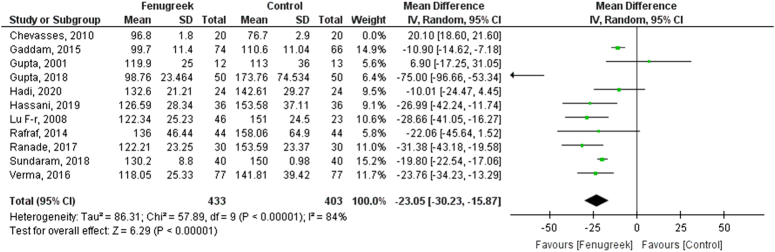
Sensitivity analysis for fasting blood sugar (FBS) for fenugreek vs. control.

**Figure 4:**
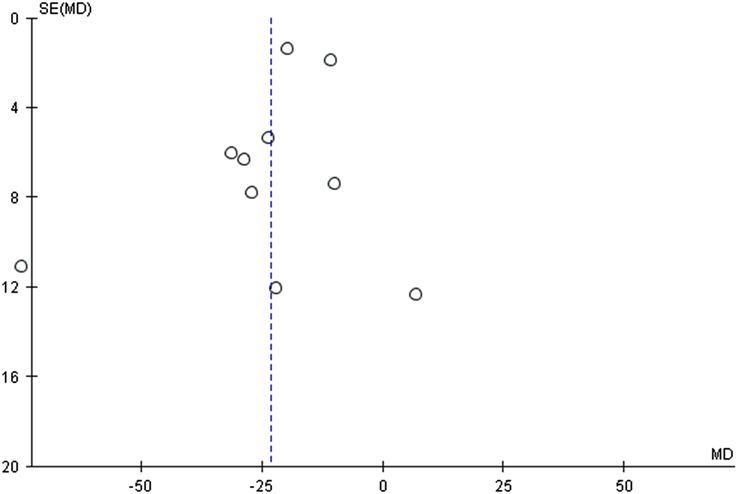
Funnel plot of fasting blood sugar (FBS) for fenugreek vs. control with sensitivity analysis.

Due to the high heterogenity, a sensitivity analysis was done in which the study Chevasses *et al*. was removed, which redcued the heterogenity from 99% to 84% (MD = −23.05 [−30.23, −5.87], *P*<0.00001, *I*^2^=84%; Figs 3, 4).

### HbA1c in T2DM patients

**Figure 5:**
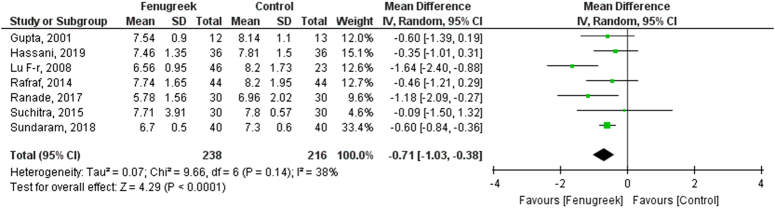
Hb1Ac for fenugreek vs. control.

**Figure 6:**
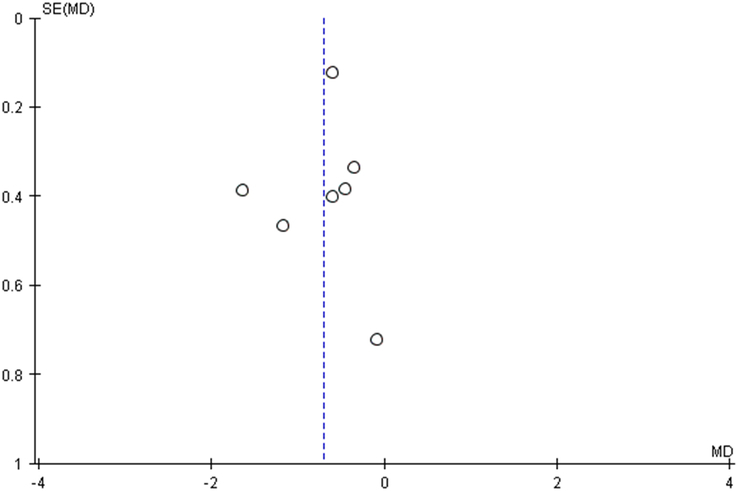
Funnel plot of Hb1Ac for fenugreek vs. control.

In our pooled analysis, 7 studies comprising 454 participants reported HbA1c, which was statistically significant. Our pooled analysis demonstrated that fenugreek effectively reduced HbA1c levels (MD = −0.71 [−1.03, −0.38], *P*=0.14, *I*^2^=38%; Figs 5, 6) when compared with vehicle in type 2 diabetic patients.

### Postprandial glucose in T2DM patients

**Figure 7:**
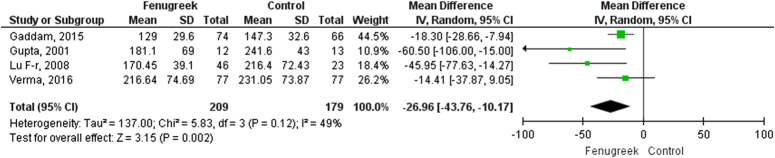
Post-prandial glucose for fenugreek vs. control.

**Figure 8:**
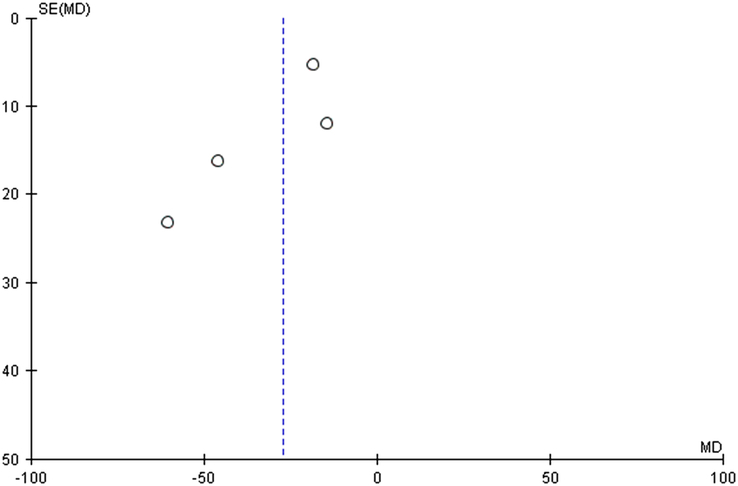
Funnel plot of postprandial glucose for fenugreek vs. control.

In our pooled analysis, 4 studies comprising 388 participants reported postprandial glucose levels, which were statistically significant. Our pooled analysis demonstrated that fenugreek effectively reduced postprandial glucose levels (MD = −26.96 [−43.76, −10.17], *P*=0.12, *I*^2^=49%; Figs 7, 8) when compared with vehicle in type 2 diabetic patients.

## Discussion

The principal findings of this meta-analysis reported a statistically significant reduction in FBS levels, HbA1c levels as well as postprandial glucose levels. Furthermore, none of the trials reported any side effects after administration of fenugreek seed at various doses^12–16^.

Contrary to an earlier reported meta-analysis by Neelakatan *et al*., conducted in 2014, none of the recently evaluated trials reported any significant difference in reduction of FBS and HbA1c at 2 and 5 g doses. The variation in the findings of the studies is due to the selection of the clinical trials; the current meta-analyses only included the trials in which the antidiabetic activity of fenugreek seed was observed in powder form; however, variation in the glycaemic profile at different doses has been attributed to the different forms of the fenugreek seed (extract, soaked in water or powder) used in various trials^11^.

Regarding the postprandial reduction in glucose levels, multiple studies have documented positive results and have supported the verdict that fenugreek seed not only works in reducing FBS but is also responsible for the reduction in postprandial glucose levels. The concept of postprandial glucose level reduction favours the hypothesis that fenugreek is an insulin secretagogue. In a study conducted by Robert *et al*., fenugreek seed powder was added to the meals of obese individuals, and glucose levels were compared. The results showed that the individuals who were given meals mixed with fenugreek powder had lesser postprandial glucose levels when compared to controls, which highlights that the addition of fenugreek may have facilitated the release of insulin from the beta islets, hence reducing the postprandial glucose^26–28^.

The exact mechanism by which fenugreek seeds reduce the glycaemic profile is not known yet. It has been documented in various animal-based trials that the antidiabetic properties of fenugreek are attributed to insulin release by the pancreatic beta cells, and due to its dyslipidemic effects, it causes decreases in insulin resistance and promotes glucose uptake by muscles^6,29,30^. As a crude herb in any form (extract, soaked in water or powder), the fenugreek seeds have shown antidiabetic effects by various actions. However, the current paradigm is shifted now to identify the potential phytoconstituents present in the fenugreek to report the exact mechanism exhibited by them^31,32^. Among the various phytoconstituents of fenugreek, 4 hydroxy isoleucine (4OHI) has been identified as a potential insulinotropic agent^33^, trigonelline has been reported as an insulin sensitizer^34^ and diosgenin has been reported as the regenerator of pancreatic beta cells^35^. Furthermore, vitexin, isovitexin^36^ and scopoletin^37^ have been evaluated for their antidiabetic effects. However, the mechanism of these and many other phytoconstituents of fenugreek is yet to be identified.

To the best of our search, the last meta-analysis that highlighted the effects of fenugreek on the glycaemic profile of humans was published in 2014^11^. After that, only five clinical trials have been conducted, which are reported in the current analysis. The strength of the current analysis included an extensive literature search and review of currently reported trials, a focused approach to identify the trials reporting FBS and HbA1c levels after administration of fenugreek in powder form only and not in combination with other herbs or standard oral hypoglycaemic drugs, and studies were selected with the presence of randomization and a controlled group. The current analysis did not report the adverse effect profile of fenugreek seed administration as the adverse effects were not examined by the authors of selected articles. Moreover, many other observers have reported it as a safer drug^28,38,39^. Despite having a safer profile, it has been documented that administration of fenugreek may result in the development of hypoglycaemia when taken with other oral hypoglycaemic agents; it can decrease the absorption of other drugs from the intestine and reduce their activity; and it can decrease the potassium level in the blood when taken with purgatives^40^. Some limitations exist in this meta-analysis. First, our results are limited due to patient heterogeneity, e.g. age, gender, body mass index and underlying comorbidities. Within our particular analysis, the heterogeneity was noted to be high. This can be attributed in large part to previous medication exposure. Given the study protocol, and the data included, it would not be pragmatic to sub-classify subjects based on previous medication exposure. Therefore, a high heterogeneity in this context should be construed with due caution. The random effects model was applied for statistical analysis because of heterogeneity. A high heterogeneity is not always followed up by regression analysis. Second, the clinical trials included had a small sample size contributing to cognitive bias. Thirdly, the duration of the studies varied greatly and was not long enough (>90 days) to correctly conclude the effect on HbA1c levels. Lastly, studies in languages other than English were not included in our analysis.

Being an easily available herbal agent and due to its promising benefits in treating diabetes and obesity, fenugreek is used by a great number of people across the globe^41,42^. Therefore, it is suggested that fenugreek should be evaluated for its adverse effect profile and drug–drug and drug–food interactions so that people may get more benefits from these traditional plants. Conversely, more randomized control trials should be conducted with larger sample size and a duration of at least 3 months to evaluate HbA1c. An efficient method of allocation concealment and transparent reporting of these techniques are necessary given the poor quality of the included trials and the potential for publication bias. The content of the fenugreek herbal product must be determined and standardized for future studies.

## Conclusion

In conclusion, this updated meta-analysis highlights the efficacy of fenugreek in lowering FBS levels, HbA1c levels and postprandial glucose levels in diabetic patients. There is still a gap of knowledge that needs to be assessed via further clinical trials on a larger sample size to evaluate the efficacy and safety profile of fenugreek in various doses and forms.

## Ethical approval

Ethics approval was not required for this systematic review and meta-analysis.

## Consent

Informed consent was not required for this systematic review and meta-analysis.

## Sources of funding

Not applicable.

## Author contribution

Conceptualization, data curation and project administration: A.A., S.S. and S.E.A.; supervision: A.A. and T.A.; formal analysis of data: S.S., S.K.F. and S.E.A.; methodology, results and software work: S.S., S.K.F., S.E.A., M.A., I.A., A.A. and T.A.; writing of the original draft: S.S., S.K.F. and S.E.A.; article review and editing: S.S., S.K.F., S.E.A., R.A., R.K.R., A.A. and T.A. All authors read and approved the final version of the manuscript.

## Conflicts of interest disclosure

Nothing to declare.

## Research registration unique identifying number (UIN)

1. Name of the registry: PROSPERO – International prospective register of systematic reviews.
2. Unique identifying number or registration ID: CRD42023443601.
3. Hyperlink to your specific registration (must be publicly accessible and will be checked): https://www.crd.york.ac.uk/prospero/display_record.php?ID=443601.

## Guarantor

Talal Almas, MD.

## Data availability statement

Not applicable.

## Provenance and peer review

Not commissioned, externally peer-reviewed.

## Supplementary Material

SUPPLEMENTARY MATERIAL
