# Supplementary figures and images for: Enhancing glycaemic control and promoting cardiovascular health: the therapeutic potential of Trigonella foenumgraecum in diabetic patients – a systematic review and meta-analysis
Source: Ann Med Surg (Lond). 2024 Jan 25;86(6):3460–7. doi: 10.1097/MS9.0000000000001750 (PMC11152803; doi:10.1097/MS9.0000000000001750)

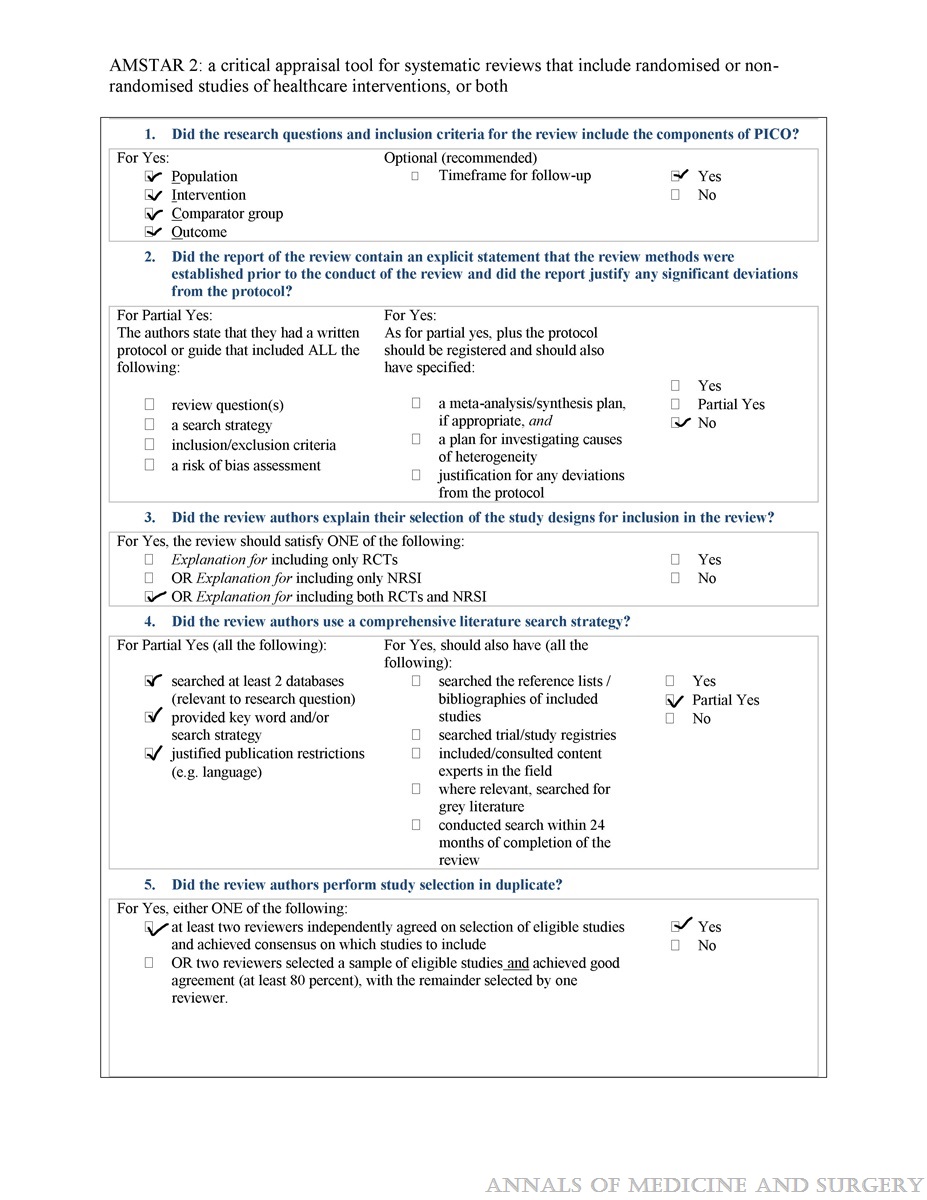


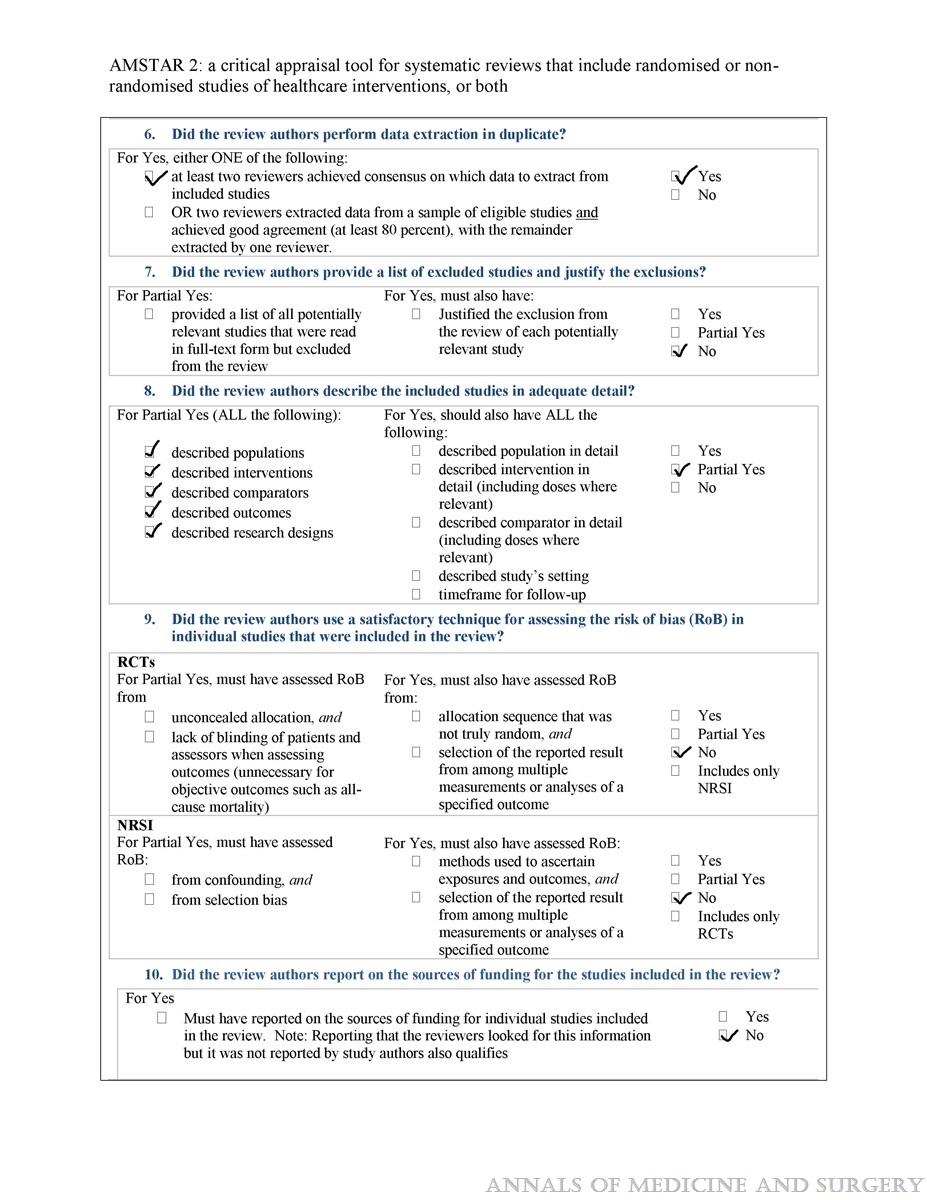


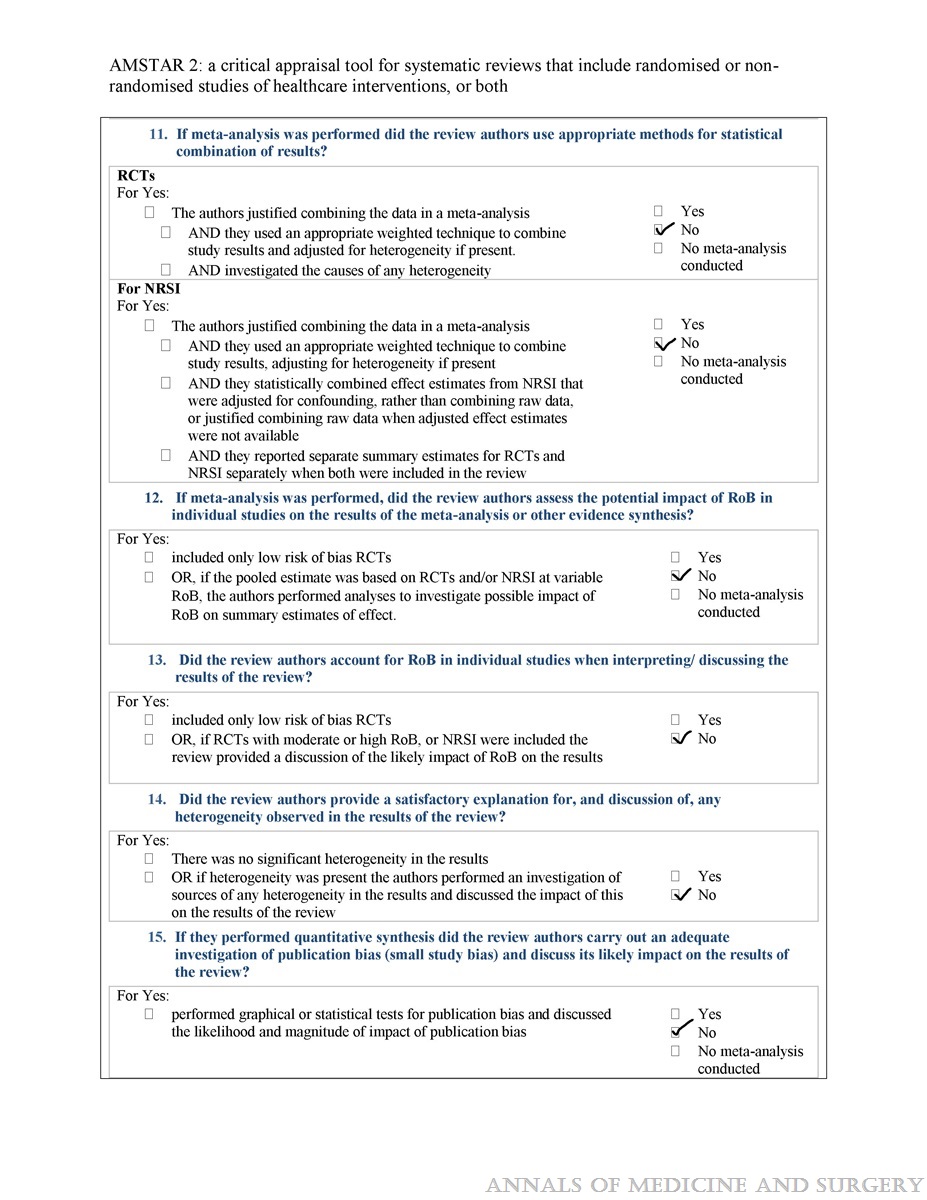


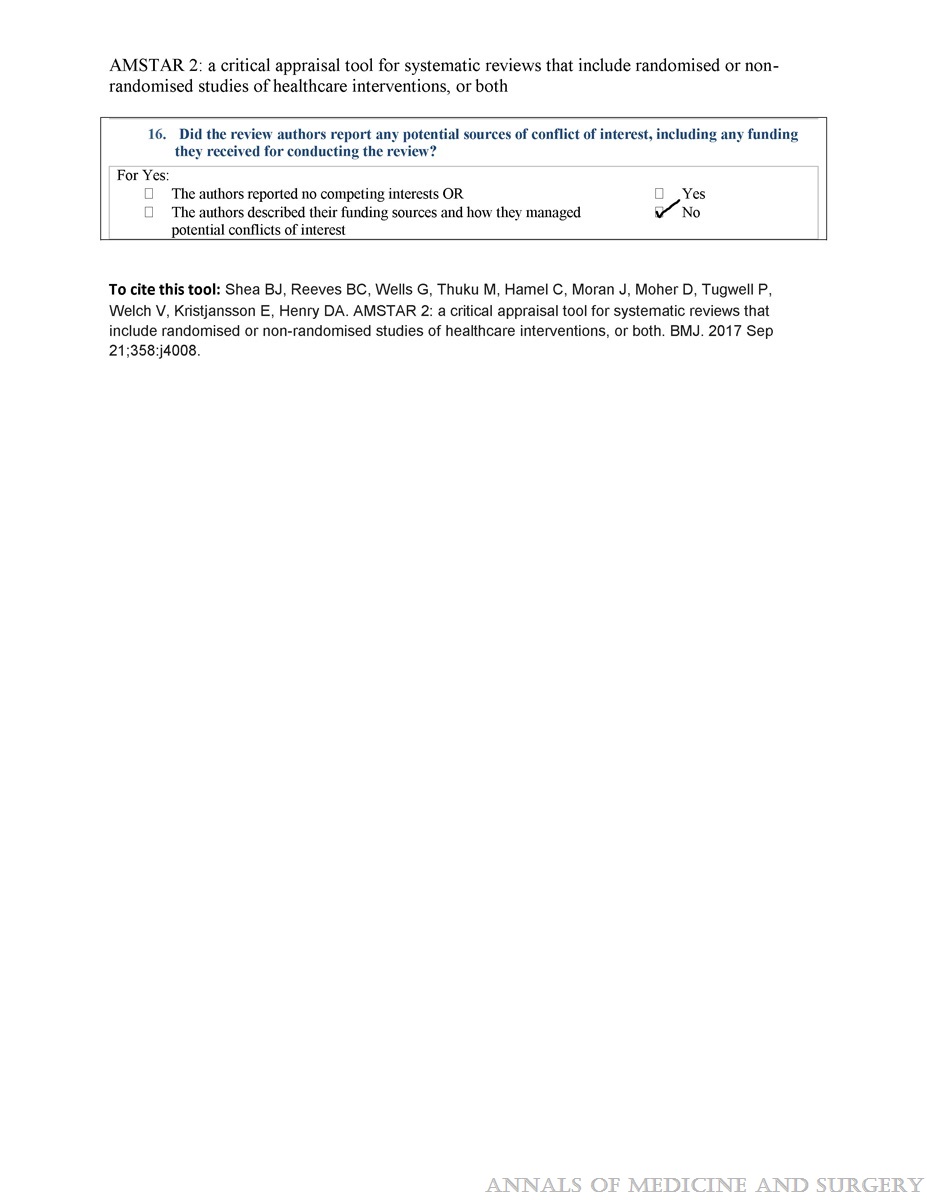

Supplement: SUPPLEMENTARY MATERIAL [file ms9-86-3460-s002.docx]
