# Supplementary material for: Enhancing glycaemic control and promoting cardiovascular health: the therapeutic potential of Trigonella foenumgraecum in diabetic patients – a systematic review and meta-analysis
Source: Ann Med Surg (Lond). 2024 Jan 25;86(6):3460–7. doi: 10.1097/MS9.0000000000001750 (PMC11152803; doi:10.1097/MS9.0000000000001750)
Supplement: SUPPLEMENTARY MATERIAL [file ms9-86-3460-s003.docx]

**SUPPLEMENTAL APPENDIX**

**Table S1**. Search strategy used in each database searched

**Figure S1.** PRISMA flow diagram for retrieval of articles

**Table S2.** Quality Assessment of studies using ROBINS-II Risk of Bias Assessment

**Table S1**. Search strategy used in database searched:

| PUBMED | ("trigonella"[MeSH Terms] OR "trigonella"[All Fields] OR "fenugreek"[All Fields] OR ("trigonella"[MeSH Terms] OR "trigonella"[All Fields] OR "trigonellae"[All Fields])) AND ("clinical trial"[Publication Type] OR "clinical trials as topic"[MeSH Terms] OR "clinical trial"[All Fields] OR ("intervention s"[All Fields] OR "interventions"[All Fields] OR "interventive"[All Fields] OR "methods"[MeSH Terms] OR "methods"[All Fields] OR "intervention"[All Fields] OR "interventional"[All Fields]) OR ("clinical trials as topic"[MeSH Terms] OR ("clinical"[All Fields] AND "trials"[All Fields] AND "topic"[All Fields]) OR "clinical trials as topic"[All Fields] OR "trial"[All Fields] OR "trial s"[All Fields] OR "trialed"[All Fields] OR "trialing"[All Fields] OR "trials"[All Fields]) OR ("therapeutics"[MeSH Terms] OR "therapeutics"[All Fields] OR "therapies"[All Fields] OR "therapy"[MeSH Subheading] OR "therapy"[All Fields] OR "therapy s"[All Fields] OR "therapys"[All Fields])) AND ("insulin"[MeSH Terms] OR "insulin"[All Fields] OR "insulin s"[All Fields] OR "insuline"[All Fields] OR "insulinic"[All Fields] OR "insulinization"[All Fields] OR "insulinized"[All Fields] OR "insulins"[MeSH Terms] OR "insulins"[All Fields] OR (("insulin"[MeSH Terms] OR "insulin"[All Fields] OR "insulin s"[All Fields] OR "insuline"[All Fields] OR "insulinic"[All Fields] OR "insulinization"[All Fields] OR "insulinized"[All Fields] OR "insulins"[MeSH Terms] OR "insulins"[All Fields]) AND ("level"[All Fields] OR "levels"[All Fields])) OR (("plasma"[MeSH Terms] OR "plasma"[All Fields] OR "plasmas"[All Fields] OR "plasma s"[All Fields]) AND ("insulin"[MeSH Terms] OR "insulin"[All Fields] OR "insulin s"[All Fields] OR "insuline"[All Fields] OR "insulinic"[All Fields] OR "insulinization"[All Fields] OR "insulinized"[All Fields] OR "insulins"[MeSH Terms] OR "insulins"[All Fields])) OR ("hypoglycaemia"[All Fields] OR "hypoglycemia"[MeSH Terms] OR "hypoglycemia"[All Fields] OR "hypoglycaemias"[All Fields] OR "hypoglycemias"[All Fields]) OR ("hyperglycaemia"[All Fields] OR "hyperglycemia"[MeSH Terms] OR "hyperglycemia"[All Fields] OR "hyperglycaemias"[All Fields] OR "hyperglycemias"[All Fields] OR "hyperglycemia s"[All Fields]) OR ("glycemia"[All Fields] OR "glycemias"[All Fields]) OR ("glucose"[MeSH Terms] OR "glucose"[All Fields] OR "glucoses"[All Fields] OR "glucose s"[All Fields]) OR (("fasted"[All Fields] OR "fasting"[MeSH Terms] OR "fasting"[All Fields] OR "fastings"[All Fields] OR "fasts"[All Fields]) AND ("blood glucose"[MeSH Terms] OR ("blood"[All Fields] AND "glucose"[All Fields]) OR "blood glucose"[All Fields])) OR ("glycosylated haemoglobin"[All Fields] OR "glycated hemoglobin"[MeSH Terms] OR ("glycated"[All Fields] AND "hemoglobin"[All Fields]) OR "glycated hemoglobin"[All Fields] OR ("glycosylated"[All Fields] AND "hemoglobin"[All Fields]) OR "glycosylated hemoglobin"[All Fields]) OR ("glycated hemoglobin"[MeSH Terms] OR ("glycated"[All Fields] AND "hemoglobin"[All Fields]) OR "glycated hemoglobin"[All Fields] OR "hba1c"[All Fields] OR "hba1cs"[All Fields]) OR (("fasted"[All Fields] OR "fasting"[MeSH Terms] OR "fasting"[All Fields] OR "fastings"[All Fields] OR "fasts"[All Fields]) AND ("serum"[MeSH Terms] OR "serum"[All Fields] OR "serums"[All Fields] OR "serum s"[All Fields] OR "serumal"[All Fields]) AND ("insulin"[MeSH Terms] OR "insulin"[All Fields] OR "insulin s"[All Fields] OR "insuline"[All Fields] OR "insulinic"[All Fields] OR "insulinization"[All Fields] OR "insulinized"[All Fields] OR "insulins"[MeSH Terms] OR "insulins"[All Fields])) OR ("diabete"[All Fields] OR "diabetes mellitus"[MeSH Terms] OR ("diabetes"[All Fields] AND "mellitus"[All Fields]) OR "diabetes mellitus"[All Fields] OR "diabetes"[All Fields] OR "diabetes insipidus"[MeSH Terms] OR ("diabetes"[All Fields] AND "insipidus"[All Fields]) OR "diabetes insipidus"[All Fields] OR "diabetic"[All Fields] OR "diabetics"[All Fields] OR "diabets"[All Fields]) OR ("diabetes mellitus"[MeSH Terms] OR ("diabetes"[All Fields] AND "mellitus"[All Fields]) OR "diabetes mellitus"[All Fields]) OR ("dyn med"[Journal] OR "dis mon"[Journal] OR "dis manag"[Journal] OR "dm"[All Fields])) |
| --- | --- |

**Table S2**: Risk of Bias Assessment of the 12 included studies using ROBINS-II tool

| Study name, year | Bias arising from the randomization process | Bias due to deviations from intended interventions | Bias due to missing data | Bias in measurement of outcomes | Bias in selection of the reported result | low/moderate/serious / critical |
| --- | --- | --- | --- | --- | --- | --- |
| **Narsingh Verma, 2016** | **Low risk** | **Low risk** | **Low risk** | **Moderate risk** | **Moderate risk** | **Moderate risk** |
| **Gopalakrishnan Sundaram, 2018** | **Low risk** | **High risk** | **Low risk** | **Moderate risk** | **Low risk** | **Moderate risk** |
| **Seyyedeh Seddigheh Hassani, 2019** | **Low risk** | **Low risk** | **Low risk** | **Low risk** | **Moderate risk** | **Low risk** |
| **Manjiri Ranade, 2017** | **Moderate risk** | **High risk** | Low risk | Low risk | Low risk | **Moderate risk** |
| **Hadi A, 2020** | **Low risk** | **Low risk** | **Low risk** | **Low risk** | **Low risk** | **Low risk** |
| **Lu F-r, 2008** | **Low risk** | **Low risk** | **Low risk** | **Low risk** | **Low risk** | **Low risk** |
| Rafraf M, 2014 | **Low risk** | **Low risk** | **Low risk** | **Low risk** | **Low risk** | **Low risk** |
| Suchitra M, 2015 | **Low risk** | **Low risk** | **Low risk** | **Low risk** | **Low risk** | **Low risk** |
| Gaddam A, 2015 | **Low risk** | **Low risk** | **Low risk** | **Low risk** | **Low risk** | **Low risk** |
| Gupta R, 2018 | **Low risk** | **Low risk** | **Low risk** | **Low risk** | **Low risk** | **Low risk** |
| Chevasus H, 2010 | **Low risk** | **Moderate risk** | **Low risk** | **Low risk** | **Low risk** | **Low risk** |
| Gupta A, 2001 | **Low risk** | **Low risk** | **Moderate risk** | **Low risk** | **Low risk** | **Low risk** |

**Figure S1. PRISMA flow diagram for retrieval of articles**

**Identification of studies via databases**

Records removed *before screening*:

Duplicate records removed (n=2)

Records identified from database searching (n =389)

**Identification**

Records excluded based on:

Title (n =151)

Abstract (n =86)

Full-text (n=79)

Records screened

(n =387)

**Screening**

Articles excluded, with reasons:

Review articles (n=47)

Cross-sectional studies (n=6)

Meta-analysis (n=6)

Full-text articles assessed for eligibility

(n=71)

**Included**

Studies included in analyses

(n=12)
